# Concurrent Ocular and Cerebral Toxoplasmosis in a Liver Transplant Patient Treated with Anti-CD40 Monoclonal Antibody

**DOI:** 10.1155/2023/5565575

**Published:** 2023-07-27

**Authors:** Roos Van Den Noortgate, Maja Kiselinova, Céline Sys, Geraldine Accou, Guy Laureys, Hans Van Vlierberghe, Frederik Berrevoet, Elke O. Kreps

**Affiliations:** ^1^Department of Ophthalmology, Ghent University Hospital, Ghent, Belgium; ^2^Department of General Internal Medicine, Ghent University Hospital, Ghent, Belgium; ^3^Department of Neurology, Ghent University Hospital, Ghent, Belgium; ^4^Department of Gastroenterology and Hepatology, Ghent University Hospital, Ghent, Belgium; ^5^Department of General and Hepatobiliary Surgery and Liver Transplantation, Ghent University Hospital, Ghent, Belgium

## Abstract

*Toxoplasma gondii*, an obligate intracellular parasitic protozoon, usually causes a mild, acute infection followed by a latent asymptomatic phase with tissue cysts or a chronic form with recurrent retinochoroiditis. However, immunocompromised patients can cause disseminated disease due to the reactivation of the latent tissue cysts or due to a primary infection. Here, we present a rare case of bilateral ocular toxoplasmosis and concurrent subacute toxoplasma encephalitis in a 70-year-old patient on anti-CD40 treatment following his liver transplant. The diagnosis was confirmed by PCR of anterior chamber fluid and brain biopsy, and no other sites of disseminated disease were detected on PET-CT. The patient has been treated with sulfamethoxazole-trimethoprim 800/160 mg with virtually complete resolution of the neurological and ocular symptoms. Iatrogenic blockade of the CD40 pathway may elicit a particular susceptibility for CNS reactivation of *T. gondii*.

## 1. Introduction

Ocular reactivation of *Toxoplasma gondii* is the leading cause of infectious retinochoroiditis worldwide, usually occurring in otherwise healthy children or young adults [1]. Central nervous system (CNS) toxoplasmosis on the other hand typically manifests in immunocompromised patients, particularly those with HIV infection, neoplastic disease, and transplant recipients [2, 3]. Secondary immune deficiency can cause the reactivation of brain cysts into cytotoxic tachyzoites, causing toxoplasmic encephalitis [3]. Iscalimab is a fully human monoclonal antibody that blocks the CD40-CD154 costimulatory pathway and has been tested in various autoimmune disorders and solid organ transplantation [4]. Here, we present a rare case of subacute cerebral and ocular toxoplasmosis occurring in a liver transplant patient during iscalimab treatment.

## 2. Case Presentation

**Figure 1:**
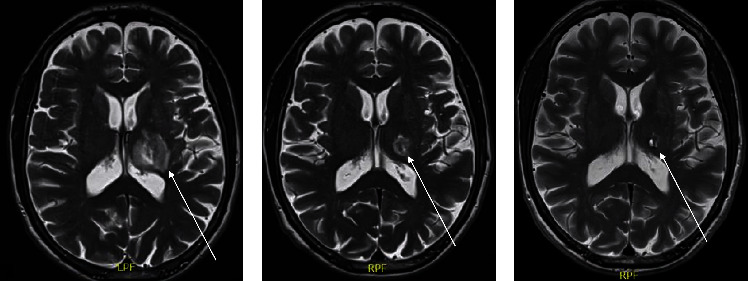
MRI imaging (T2 images, sagittal view) at presentation (a) and during follow-up (b) 5 weeks of TMP-SFX twice daily; (c) 20 weeks follow-up, of which 6 weeks of TMP-SFX 4 times a day and 14 weeks twice daily. Gradual improvement of the lesion was seen during treatment (highlighted by arrow).

**Figure 2:**
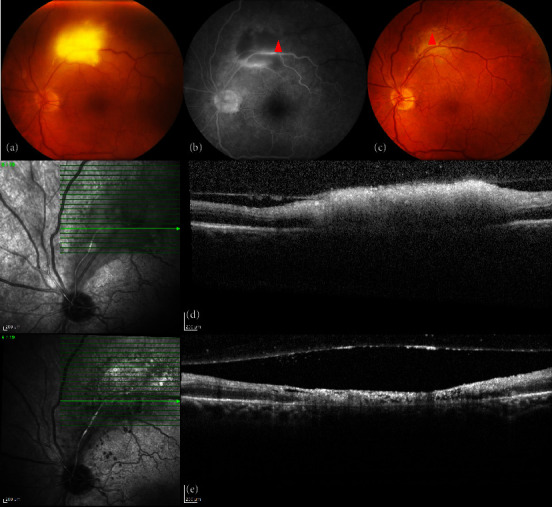
Ocular features at presentation and during follow-up. (a) The left eye showed a white retinitis lesion along the superotemporal arcade (colour fundus picture). (b) Adjacent Kyrieleis arteritis (arrow) and ischaemia on fluorescein angiography at first visit. (d) Optical coherence tomography demonstrates the full-thickness retinitis (arrow) with limited adjacent vitritis. (c) With treatment, the lesion gradually became atrophic ((e) 2 months follow-up) and vascular supply was restored via a collateral artery.

A 70-year-old male was referred to the Neurology Department for complaints of right-sided upper extremity muscle weakness and gait instability for 4 weeks. His medical history included hepatitis *C*-related liver cirrhosis, for which he had an orthotopic liver transplantation one year prior. He was currently on iscalimab treatment, an anti-CD40 monoclonal antibody 300 mg administered subcutaneously every 2 weeks, to prevent transplant rejection, and oral valganciclovir 450 mg once daily to avoid CMV reactivation. During the first 4 months following his transplant, he had also been treated with systemic corticosteroids and mycophenolate mofetil. The neurologic assessment revealed a right-sided spastic paresis with hyperreflexia, hypertonia, and ankle clonus, for which urgent cranial MRI imaging was requested. A 1.5 cm contrast-enhancing lesion in the left basal ganglia area was detected with significant perilesional oedema and limited mass effect with narrowing of the trigonum of the left lateral ventricle (Figure 1(a)). He was also seen at the Ophthalmology Department on the day of MRI imaging because of a one-week history of left-sided visual loss. He had no prior ocular history. Six days before, his local ophthalmologist had commenced Pred Forte eye drops 3 times a day to the left eye and systemic acyclovir (800 mg 5 times a day) because of presumed herpetic uveitis. At the ocular exam in our department, visual acuity was 1.0 in the right eye and 0.7 in the left (decimal Snellen). In the left eye, 0.5+ cells in the anterior chamber and a very mild vitreous haze (BIO score 0.5) were seen, whereas the right eye showed no inflammatory activity. Intraocular pressure was normal (<21 mmHg) in both eyes. Fundus examination disclosed a small midperipheral white retinitis lesion in the right eye and a larger retinal necrotic lesion in the left eye with associated Kyrieleis arteritis (Figure 2(a)). No old chorioretinal scars were seen in either eye. An anterior chamber tap was performed for PCR analysis of herpetic species and *Toxoplasma gondii*, and sulfamethoxazole-trimethoprim 800/160 mg (TMP-SFX) was empirically initiated twice daily, along with continuation of topical steroids and oral antiviral treatment. Four months prior to presentation, serologic testing had shown positive anti-*Toxoplasma* IgG and negative IgM antibodies.

PCR analysis of the ocular fluid subsequently came back positive for *Toxoplasma gondii*, and systemic TMP-SFX treatment was continued (oral acyclovir was stopped). Further neurologic workup was simultaneously performed, included MRI spectroscopy—which confirmed a contrast-enhancing lesion with central necrosis in the left thalamus, suggestive of CNS lymphoma—and a lumbar puncture. Liquor analysis showed normal biochemical and cellular composition, no signs of malignancy, and negative PCR for viral species and *T. gondii*. A full-body PET-CT was performed, which did not highlight any other metabolically active lesions.

The neurologic state of the patient remained stable in the weeks following presentation and a cranial biopsy was subsequently performed, which showed bradyzoites with no evidence of malignancy and immunohistochemical staining was positive for toxoplasmosis. Considering these findings, iscalimab treatment was switched to everolimus, and the dose of sulfamethoxazole-trimethoprim 800/160 mg (TMP-SFX) was increased to four times a day for six weeks. Follow-up cranial MRI imaging showed a gradual decrease in the thalamic lesion and the surrounding oedema. The retinitis lesions in both eyes gradually became atrophic (Figure 2(c)). At 6-month follow-up, marked improvement of his neurologic state was noted with mild residual right-sided hyperreflexia and atactic gait and stable ocular findings. Systemic TMP-SFX 800/160 mg twice daily was continued as ongoing prophylaxis.

## 3. Discussion

In this 70-year-old liver transplant patient, *Toxoplasma gondii* proliferation from a past latent infection occurred in the ocular tissue of both eyes and the left thalamus, resulting in the subacute occurrence of cerebral and ocular toxoplasmosis whilst on immunomodulatory treatment. Muscle, brain, and retinal tissue are preferential sites of *T. gondii* latency following primary infection. In immunocompetent patients, usually, young people, reactivation in the retina leads to a recurrent, characteristic retinochoroiditis adjacent to an old scar with marked vitritis which is self-limited in nature. In immunodeficient patients, atypical larger and/or bilateral lesions can be seen with varying degrees of surrounding inflammation, necessitating intraocular fluid analysis to confirm the diagnosis. The intracellular protozoan can also manifest outside the retina in immunocompromised patients, most commonly in the central nervous system. Toxoplasmic encephalitis usually presents in the setting of advanced immunosuppression [2], typically with multiple ring-enhancing lesions and surrounding oedema on cranial imaging in predominantly the basal ganglia, and is one of the most lethal manifestations of *T. gondii* infection [5].

Toxoplasmic reactivation is a rare but recognized phenomenon following noncardiac solid organ transplantation. It most commonly presents in the first 180 days posttransplant with disseminated disease (in 42% of cases), defined as 2 or more organs involved, and features nonspecific presenting symptoms including headache, confusion, fever, focal neurologic deficits, psychomotor retardation, and disturbed consciousness [6]. Simultaneous cerebral and (bilateral) ocular toxoplasmosis is an unusual presentation, particularly in a 70-year-old patient without evidence of prior toxoplasmic retinochoroiditis (lack of chorioretinal scarring).

The immune response to toxoplasmosis involves a complex interaction of innate proinflammatory, CD4+, CD8+, and *B* lymphocyte mediators [7]. Interferon-*γ* has long been recognized as an important cytokine against *T. gondii* infection and replication in host cells [8]. Recent research also points to a significant role of CD40 and CD154 (=CD40 ligand) as the CD40-CD154 pathway regulates interleukin-12 (IL-12) production and elicits degradation of *T. gondii* in macrophage and microglial cells (hematopoietic cells) through the autophagy pathway [9, 10]. Particularly in nonhematopoietic infected cells such as human retinal pigment epithelial cells and microvascular brain endothelial cells, CD40 stimulation leads to an activation of toxoplasmacidal activity through the autophagy pathway [9, 10]. Inhibition of the CD40/CD154 pathway may therefore potentiate the reactivation of latent cysts in cerebral and ocular tissue with consequent parasite proliferation and tissue damage. The role of the CD40/CD154 pathway in eliciting cell-mediated immunity in the defense against intracellular infection has also been demonstrated by a case of latent tuberculosis reactivation in a renal transplant patient on iscalimab [11]. This patient's age and recent systemic corticosteroids and mycophenolate mofetil are also likely to have contributed to his immunodeficient state.

We present a rare case of bilateral ocular toxoplasmosis and concurrent toxoplasma encephalitis in a 70-year-old patient on anti-CD40 treatment following his liver transplant. An excellent neurologic and ocular outcome was seen with systemic TMP-SFX treatment, which aligns with recent data on its efficacy in cerebral toxoplasmosis [12]. Iatrogenic blockade of the CD40/CD154 pathway may increase the risk of reactivation of *T. gondii* in cerebral and ocular tissue.

## Data Availability

The data used to support the findings of this study are included within the article.
